# Canadian Creutzfeldt-Jakob disease incidence remained stable during the coronavirus disease (COVID-19) pandemic

**DOI:** 10.3389/fneur.2025.1729083

**Published:** 2025-12-19

**Authors:** Jessy A. Slota, Dobrila Todoric, Vanessa Bergeron, Kristen Avery, Clark Phillipson, Dominic M. S. Kielich, Jennifer Myskiw, Lise Lamoureux, Kathy Frost, Sharon L. R. Simon, Ben A. Bailey-Elkin, Stephanie A. Booth

**Affiliations:** 1 Mycobacteriology, Vector-Borne and Prion Diseases Division, National Microbiology Laboratory, Public Health Agency of Canada, Winnipeg, MB, Canada; 2 Infectious Diseases and Vaccination Programs Branch, Public Health Agency of Canada, Ottawa, ON, Canada; 3 Department of Medical Microbiology and Infectious Diseases, Faculty of Health Sciences, University of Manitoba, Winnipeg, MB, Canada

**Keywords:** coronavirus disease, Creutzfeldt-Jakob disease, diagnostics, prion disease, SARS-CoV-2, surveillance

## Abstract

**Introduction:**

Healthcare disruptions imposed by the coronavirus disease (COVID-19) pandemic and possible biological links between SARS-CoV-2 and prion misfolding might influence the prevalence or characteristics of Creutzfeldt-Jakob Disease (CJD). This report investigates the potential impact of the COVID-19 pandemic on Canadian CJD diagnostics and surveillance from 2016–2025.

**Methods:**

Canada-wide CJD diagnostic findings from end-point quaking induced conversion (EP-QuIC) cerebrospinal fluid (CSF) assays were compared across three periods: pre- (2016-01-29 – 2020-02-28), during (2020-03-01 – 2022-09-30), and post-COVID-19 (2022-10-01 – 2025-09-29). Presented are incidence rates and distributions of biomarker abundances, case demographics, CJD molecular subtypes, and disease durations.

**Results:**

While EP-QuIC test submissions increased during the pandemic, CJD incidence was unaltered and not associated with SARS-CoV-2 incidence. Demographics, disease durations, and molecular subtypes of sporadic CJD (sCJD) were largely consistent across periods, although a higher proportion of females were tested during COVID and the prevalence of sCJD MV1 declined post-COVID.

**Conclusion:**

CJD prevalence and characteristics remained stable during COVID-19 despite increased EP-QuIC test submissions. These findings verify that CJD surveillance in Canada remained vigilant during the pandemic and highlight the value of EP-QuIC CSF testing for comprehensive CJD monitoring.

## Introduction

Creutzfeldt-Jakob disease (CJD) is a rare, fatal, and transmissible neurodegenerative disorder caused by misfolded prion proteins accumulating in the brain (1). While human prion diseases can be inherited or acquired, most cases are sporadic (sCJD) with an annual incidence of 1–2 per million (2).

CJDs clinical presentation often mimics other neurological disorders, necessitating accurate diagnostic tools. Modern ante-mortem CJD testing is based on quaking induced conversion assays (QuIC) that detect prion seeding activity in endpoint (EP) and real-time (RT) formats (3–8). In Canada, CJD diagnosis and surveillance rely on EP-QuIC testing of cerebrospinal fluid (CSF), which has proven highly sensitive and specific (9, 10). Consequently, a neuropsychiatric syndrome with a positive QuIC test supports a diagnosis of probable CJD and this definition is implemented in clinical and surveillance settings (4, 5). QuIC-mediated surveillance also informs case investigations to help identify atypical cases and iatrogenic exposures, which can be further augmented by examining clinical histories, neuropathology, and PrP^Sc^ molecular subtyping (11).

Healthcare disruptions, such as those caused by the coronavirus disease (COVID-19) pandemic, might impede CJD case identification and surveillance because lockdowns, strained medical infrastructure, or reduced access to health services may impact testing availability. Additionally, biological aspects of SARS-CoV-2 infection such as cytokine storm (12), neuroinflammation (13), amyloid-*β* aggregation (14, 15), and the amyloidogenic properties of SARS-CoV-2’s spike protein (16, 17) might influence prion misfolding or disease progression. Although studies from the United Kingdom (18), Germany (19, 20), and Australia (21) found no link between COVID-19 and CJD prevalence, we previously noted a decline in Canadian EP-QuIC positivity rates that seemed to coincide with pandemic (10).

To investigate whether COVID-19 influenced Canadian CJD diagnostics or surveillance, we compared CJD diagnostic testing across three periods: pre-COVID, COVID, and post-COVID. We assessed CSF test submission volumes, CJD incidence (estimated from positive EP-QuIC tests), patient demographics (age and sex), *PRNP* codon 129 genotype, and biomarker levels (14-3-3 gamma and total Tau proteins). To identify potential shifts in disease presentation, we also examined sCJD subtype distributions and disease durations in those cases for which information was available. While CSF test submissions increased during COVID, CJD incidence remained stable. Also noted was a higher proportion of females tested during COVID and a post-pandemic decline in MV1 subtype prevalence. These results demonstrate the contribution of EP-QuIC testing to CJD surveillance, which remained consistent throughout the pandemic.

## Methods

### Setting, population, and case definitions

This retrospective study analyzed data from 3,001 individuals that underwent EP-QuIC CSF testing in Canada between January 29th, 2016, when EP-QuIC was first implemented, and the study cut-off of September 29th, 2025. Clinicians across Canada typically submit CSF specimens collected from live patients presenting with rapid neurological decline. Among the 3,001 CSF specimens included in this study, 641 were EP-QuIC-positive. Patients were grouped into three time intervals based on the date of EP-QuIC testing: pre-COVID (2016-01-29 – 2020-02-28), COVID (2020-03-01 – 2022-09-30), and post-COVID (2022-10-01 – 2025-09-29). The COVID period was defined by the detection of COVID-19 in Canada to the lifting of all pandemic related restrictions.

**Figure 1:**
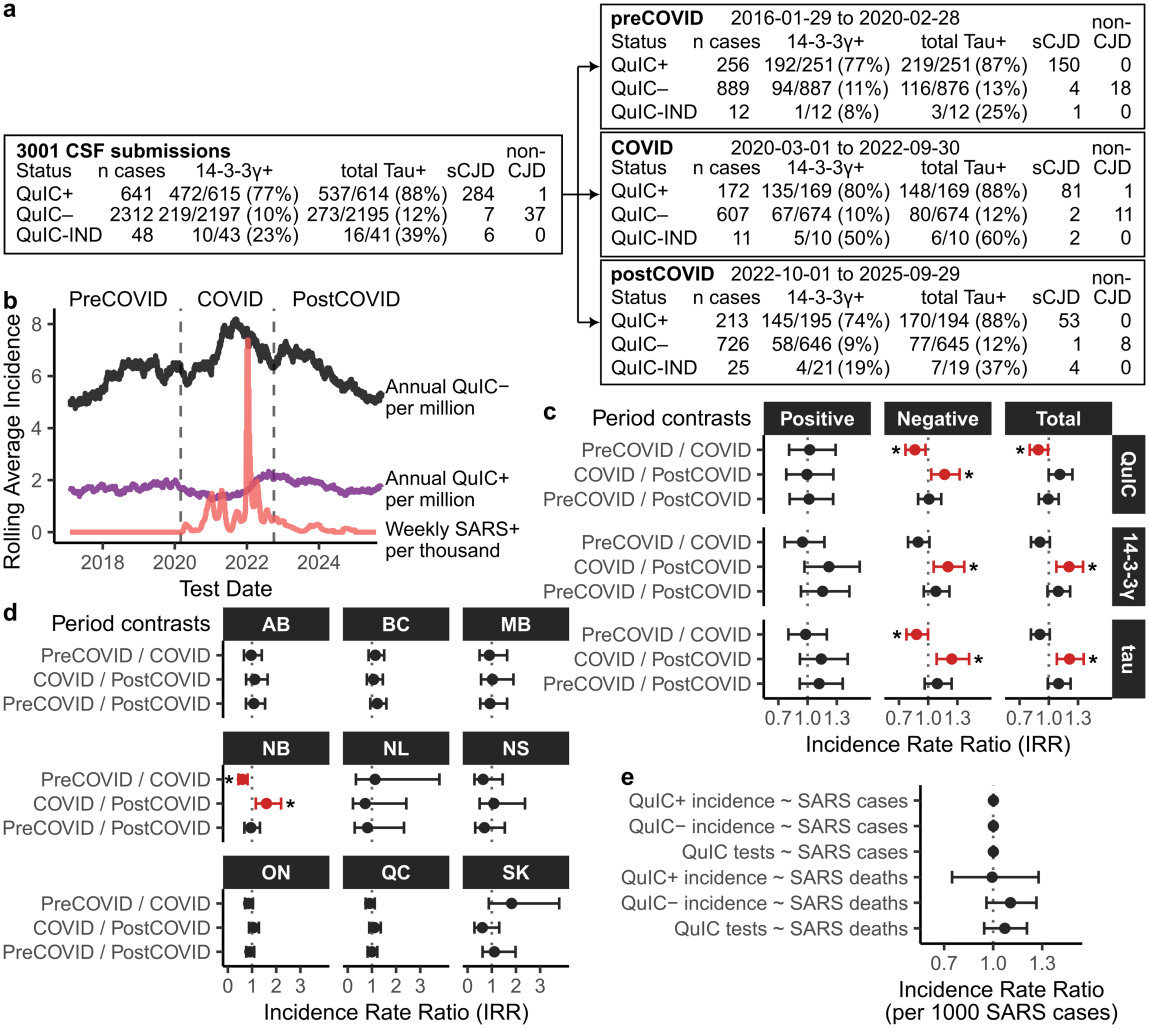
Comparing EP-QuIC results and test submissions in Canada across pre-COVID, COVID, and post-COVID periods. **(a)** Summary of EP-QuIC-tested cases across time period cohorts, stratified by EP-QuIC result and annotated with biomarker positivity rates, numbers of confirmed sCJD cases associated with a molecular subtype, and numbers of confirmed non-CJD cases. **(b)** Longitudinal trends in EP-QuIC testing and SARS-CoV-2 case incidence from 2016–2025. EP-QuIC test incidences (annual positive and negative results per million population) were smoothed using 365-day rolling averages; SARS-CoV-2 incidence (weekly cases per thousand population) was smoothed using 7-day rolling averages. **(c)** Incidence rate ratios from pairwise comparisons of CSF test incidence, including EP-QuIC (positive, negative, and total), 14-3-3 gamma, and total Tau, across time periods using Poisson regression. **(d)** Provincial incidence rate ratios from pairwise comparisons of total EP-QuIC test submissions between time periods using Poisson regression. **(e)** Incidence rate ratios from modeling EP-QuIC test incidences (positive, negative, and total) in relation to SARS-CoV-2 incidences (cases and deaths, scaled per 1,000) using Poisson regression. Estimates are shown with 95% confidence intervals. * *p* < 0.05. Pre-COVID = 2016-01-29 – 2020-02-28, COVID = 2020-03-01 – 2022-09-30, post-COVID = 2022-10-01 – 2025-09-29. QuIC-IND = Indeterminate by EP-QuIC, ON = Ontario, QC = Quebec, BC = British Columbia, AB = Alberta, MB = Manitoba, SK = Saskatchewan, NS = Nova Scotia, NB = New Brunswick, NL = Newfoundland and Labrador.

Figure 1a summarizes the EP-QuIC status of cases assigned to each time period cohort, along with 14-3-3 gamma and total tau biomarker positivity rates. A subset of these EP-QuIC-tested cases were confirmed as CJD/non-CJD via post-mortem evaluation, which are indicated in Figure 1a as the number of confirmed sCJD cases with complete molecular subtype information and the number of confirmed non-CJD cases. One false positive and seven false negatives by EP-QuIC were included in this cohort.

### Data sources

CJD CSF testing and post-mortem molecular subtyping were performed by the Prion Diseases Section at the National Microbiology Laboratory (Winnipeg, MB) and are described below. Affiliated data included EP-QuIC (3,001/3001), 14-3-3 gamma (2,855/3001), and total Tau (2,850/3001) CSF test result (positive, negative, or indeterminate), 14-3-3 gamma (2,841/3001) and total Tau (2,840/3001) biomarker abundances, PrP^Sc^ glycotype (333/3001), codon-129 genotype (495/3001), age at testing (2,994/3001), province (2,996/3001), biological sex (2,893/3001). Disease durations were available for 460 sCJD cases that could be linked with dates of symptom onset and death. External data sources included age-stratified population data from Statistics Canada^1^ and SARS-CoV-2 case numbers from: https://health-infobase.canada.ca/respiratory-virus-surveillance/.

### CSF testing

CSF specimens were tested using EP-QuIC as previously described (10). Briefly, 15 μL of CSF was added to 100 μL reactions containing PBS, 100 μg/mL recombinant full-length hamster PrP (residues 23–231), 160 mM NaCl, 10 μM EDTA, and 10 μM Thioflavin T (ThT). Triplicate reactions were run in 96-well plates, shaken at 900 rpm (90 s shaking/30 s rest) for 66 h at 42 °C using an Eppendorf Thermomixer C. ThT fluorescence (excitation 450 nm; emission 480 nm) was measured at 0 h and 66 h using a FLUOstar OMEGA plate reader. Positive reactions were defined by a ≥ 4-fold increase over the baseline. EP-QuIC positive and negative CSF specimens were defined by 3/3 and 0/3 positive replicates, respectively. Specimens with 1/3 or 2/3 positive replicates were re-run as a dilution series with 7.5, 15, and 30 μL of CSF, and classified as indeterminate if this dilution series did not provide a positive or negative result.

CSF levels of 14-3-3 gamma protein and total tau protein biomarkers were measured using the Circulex 14-3-3 Gamma (cat. no. CY8082) and Innotest hTau ELISA (cat. no. 81579) kits, respectively. Specimens with ≥ 20,000 absorbance units of 14-3-3 gamma or ≥ 976 pg./mL total Tau were classified as biomarker-positive.

### sCJD molecular subtyping

Complete subtype data were available for 297 EP-QuIC tested sCJD cases that underwent post-mortem PrP^Sc^ glycotyping and *PRNP* gene sequencing. PrP^Sc^ glycotypes (Type 1, Type 2, or Mixed) were determined by western blotting proteinase K digested brain homogenates using the 3F4 antibody (22). Molecular subtypes were assigned based on glycotype and *PRNP* codon 129 genotype (MM, MV, or VV).

### Data analysis and statistics

All statistical analyses were performed in R using the dplyr, tidyr, emmeans, nnet, survival, and ggplot2 packages.

Age-adjusted incidences of positive, negative, and total CSF tests were calculated as previously described (10). Cases were binned into age strata (0–17, 18–24, 25–44, 45–64, ≥ 65 years) and weighted using Canada’s 2011 population as the reference. Incidences (per million person years) were calculated by summing weighted cases across age strata for each study period. Incidence rates were compared using log-linked Poisson regression with province and age group as co-variates and log(person-years) as an offset. Estimated marginal means were used to compute incidence rate ratios (IRRs) for pairwise comparisons between periods. EP-QuIC test incidence rates were also modeled in relation to SARS-CoV-2 cases and deaths using log-linked Poisson regression, with a province as a co-variate and offset by log(person-years). These models did not include age-adjustment since ages were not available for SARS-CoV-2 cases. Incidence rate ratios were determined from model coefficients.

Biomarker levels were analyzed using Gamma generalized linear models (GLMs), age distributions using Gaussian GLM, codon 129 genotype and sCJD molecular subtype distributions using multivariate logistic regression, and biological sex distributions using logistic regression. EP-QuIC result (positive or negative) was included as an interaction term, and cases with an indeterminate EP-QuIC result were not included in these models. Covariates included province, age group, codon 129 genotype, and biological sex where appropriate. Pairwise comparisons between study periods were performed using estimated marginal means.

CJD disease duration was calculated from symptom onset to death for 460 sCJD cases with complete date information. Kaplan–Meier survival curves were used to visualize durations across study periods. Between-period comparisons were performed using Cox proportional hazards ratios, with age group and sex as covariates, and stratified by molecular subtype. Analyses were right-censored at the post-COVID maximum duration of 874 days.

## Results

### Total CJD CSF test submissions increased during the COVID period

To investigate whether CJD prevalence or testing patterns were altered during the pandemic, we compared CSF test results and submission levels across three time intervals: pre-COVID (2016-01-29 – 2020-02-28), COVID (2020-03-01 – 2022-09-30), and post-COVID (2022-10-01 – 2025-09-29). The COVID period was defined as March 2020 – September 2022, spanning from first detection of SARS-CoV-2 in Canada and the immediate implementation of pandemic-related restrictions through to their complete lifting in late 2022. Figure 1b shows EP-QuIC testing levels and SARS-CoV-2 incidences in relation to these externally defined milestones, which were selected to reflect nation-wide healthcare disruptions independently of CJD testing patterns.

Table 1 presents national and provincial CSF test submission incidences. Poisson regression analysis revealed a significant elevation in EP-QuIC test submissions during the COVID period compared to the pre-COVID interval (Figure 1c). Test submission levels before and after the pandemic were nearly identical (Table 1), suggesting the increase was specific to the COVID era.

**Table 1:** Canada-wide and provincial incidences of total CSF test submissions before, during, and after the COVID-19 period (per million person years).

Province	Pre-COVID [95% CI]	COVID [95% CI]	Post-COVID [95% CI]
Canada	7.02 [6.33–7.81]	7.94 [7.07–8.98]	7.05 [6.31–7.91]
ON	5.58 [4.65–6.80]	6.57 [5.42–8.14]	6.19 [5.19–7.54]
QC	7.76 [6.33–9.67]	8.50 [6.81–10.97]	7.78 [6.25–9.94]
BC	8.00 [6.23–10.58]	7.18 [5.22–10.40]	6.51 [4.77–9.26]
AB	5.91 [4.17–8.63]	6.23 [4.20–9.76]	5.51 [3.79–8.41]
MB	6.66 [3.80–12.60]	7.27 [3.91–15.36]	7.34 [4.01–14.51]
SK	8.16 [4.58–15.37]	4.34 [1.76–12.77]	7.60 [3.59–16.23]
NS	3.26 [1.40–9.69]	5.57 [2.24–15.78]	4.66 [1.97–13.00]
NB	28.88 [19.88–43.36]	52.16 [35.37–77.08]	29.98 [19.99–46.81]
NL	3.74 [0.99–15.10]	3.41 [0.70–20.07]	4.83 [1.57–19.72]
PE	1.59 [0.04–32.56]	4.16 [0.11–50.80]	0.00 [0.00–34.43]

Pre-COVID = 2016-01-29 – 2020-02-28, COVID = 2020-03-01 – 2022-09-30, post-COVID = 2022-10-01 – 2025-09-29, CI = confidence interval, ON = Ontario, QC = Quebec, BC = British Columbia, AB = Alberta, MB = Manitoba, SK = Saskatchewan, NS = Nova Scotia, NB = New Brunswick, NL = Newfoundland and Labrador, PE = Prince Edward Island.

Many provinces mirrored the Canada-wide trend of elevated total CSF test submissions during COVID followed by a return to baseline levels post-pandemic (Table 1). However, this trend reached significance only in New Brunswick (Figure 1d), which submitted many CSF samples despite having a comparatively small population. Among other provinces, CSF submission levels were comparable before, during, and after the COVID period as no significant differences were detected.

Despite some inter-provincial variation, the nation-wide average of increased CSF testing during the pandemic (Figure 1c) likely explains the previously observed decline in EP-QuIC positivity rates (10).

### CJD incidence remained stable before, during, and after COVID

Since EP-QuIC test submissions increased during the COVID period, we next examined whether CJD incidence was affected. Annual age-adjusted incidences of positive and negative EP-QuIC and biomarker CSF tests were compared across the pre-COVID, COVID, and post-COVID intervals (Table 2). While negative EP-QuIC tests increased during COVID, positive test levels remained similar across all three periods with no significant differences detected (Figure 1c).

**Table 2:** Canada-wide annual incidences of positive and negative CSF test results before, during, and after the COVID-19 period (per million person years).

Period	Positive test incidence [95% CI]	Negative test incidence [95% CI]
EP-QuIC		
Pre-COVID	1.53 [1.25–1.92]	5.41 [4.80–6.13]
COVID	1.49 [1.19–1.97]	6.35 [5.56–7.31]
Post-COVID	1.53 [1.23–1.97]	5.34 [4.69–6.11]
14-3-3 gamma		
Pre-COVID	1.73 [1.41–2.15]	5.18 [4.59–5.88]
COVID	1.83 [1.47–2.37]	5.88 [5.12–6.80]
Post-COVID	1.49 [1.20–1.93]	4.80 [4.20–5.54]
Total tau		
Pre-COVID	2.03 [1.69–2.48]	4.87 [4.30–5.55]
COVID	2.07 [1.68–2.63]	5.64 [4.89–6.55]
Post-COVID	1.80 [1.47–2.28]	4.46 [3.88–5.17]

Pre-COVID = 2016-01-29 – 2020-02-28, COVID = 2020-03-01 – 2022-09-30, post-COVID = 2022-10-01 – 2025-09-29, CI = confidence interval.

To further investigate whether CJD incidence or testing patterns were linked with SARS-CoV-2, we fit Poisson regression models to monthly incidences of QuIC tests and SARS-CoV-2 cases. Predictors were scaled to reflect changes per 1,000 SARS-CoV-2 cases or deaths. The models yielded incidence rate ratios (Figure 1e) that showed no significant associations between SARS-CoV-2 and CJD test submissions or positive diagnoses. Overall, these findings suggest that CJD incidence remained stable throughout the pandemic.

### Population demographics were largely consistent across time periods

Increased negative EP-QuIC tests during the COVID period may reflect changes in clinical rationales for testing, potentially linked to shifts in demographics of individuals tested for CJD. To explore this, we analyzed biomarker abundances and available demographic (age and biological sex) and genetic (*PRNP* codon 129 genotype) data across the pre-COVID, COVID, and post-COVID intervals.

**Figure 2:**
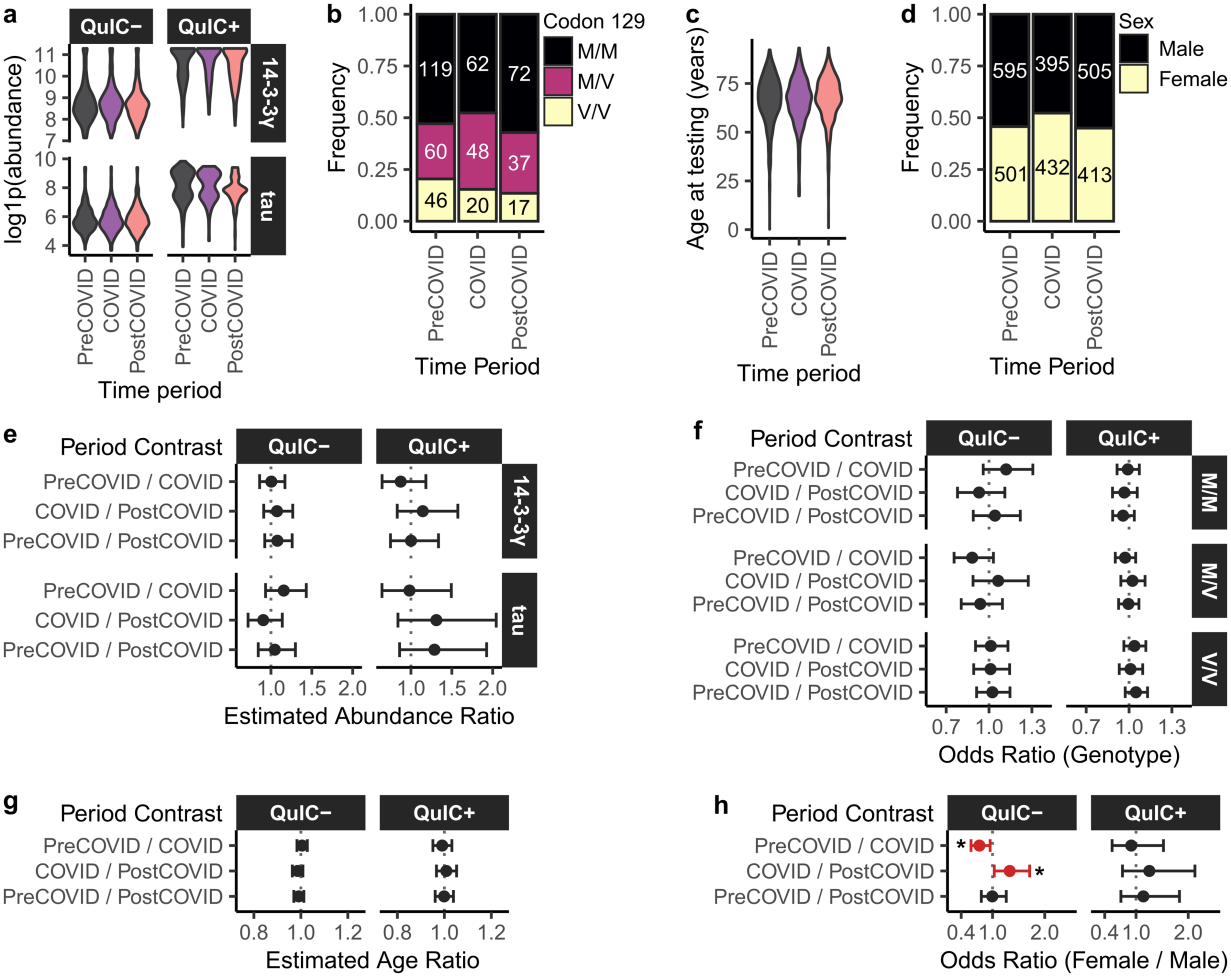
Biomarker abundances and demographics of the EP-QuIC-tested population in Canada across pre-COVID, COVID, and post-COVID periods. Shown are distributions of **(a)** 14-3-3 gamma and total Tau biomarker abundances stratified by EP-QuIC status, **(b)** codon 129 genotype, **(c)** age at testing, and **(d)** biological sex. Panels **(e–h)** show pairwise comparisons across study periods using generalized linear models: **(e)** biomarker abundances (Gamma GLM), **(f)** codon 129 genotype (multivariate logistic regression), **(g)** age at testing (Gaussian GLM), and **(h)** biological sex (binomial logistic regression). Estimates are shown with 95% confidence intervals. * *p* < 0.05. Pre-COVID = 2016-01-29 – 2020-02-28, COVID = 2020-03-01 – 2022-09-30, post-COVID = 2022-10-01 – 2025-09-29.

Levels of 14-3-3 gamma and total Tau biomarkers in EP-QuIC positive and negative CSF specimens were comparable across all three periods, as examined via a Gamma GLM model (Figures 2a,e). This suggests that population-level exposure to SARS-CoV-2 did not influence these markers of neuronal damage in this cohort. Similarly, no significant differences were observed in codon-129 genotype distributions (multinomial logistic regression, Figures 2b,f), or age-at-testing (Gaussian GLM, Figures 2c,g) across the study periods. However, multinomial logistic regression revealed that the proportion of females among EP-QuIC negative submissions significantly increased during the pandemic compared to the pre- (*p* = 0.014) and post-COVID (*p* = 0.022) intervals (Figures 2d,h).

Overall, aside from a temporary increase in female test submissions during the pandemic, population demographics among individuals tested for CJD remained stable. A more detailed understanding of the criteria used for CJD test submissions during COVID would require access to clinical information associated with submitted CSF specimens.

### sCJD subtype distributions and disease durations were largely unchanged across study periods

**Figure 3:**
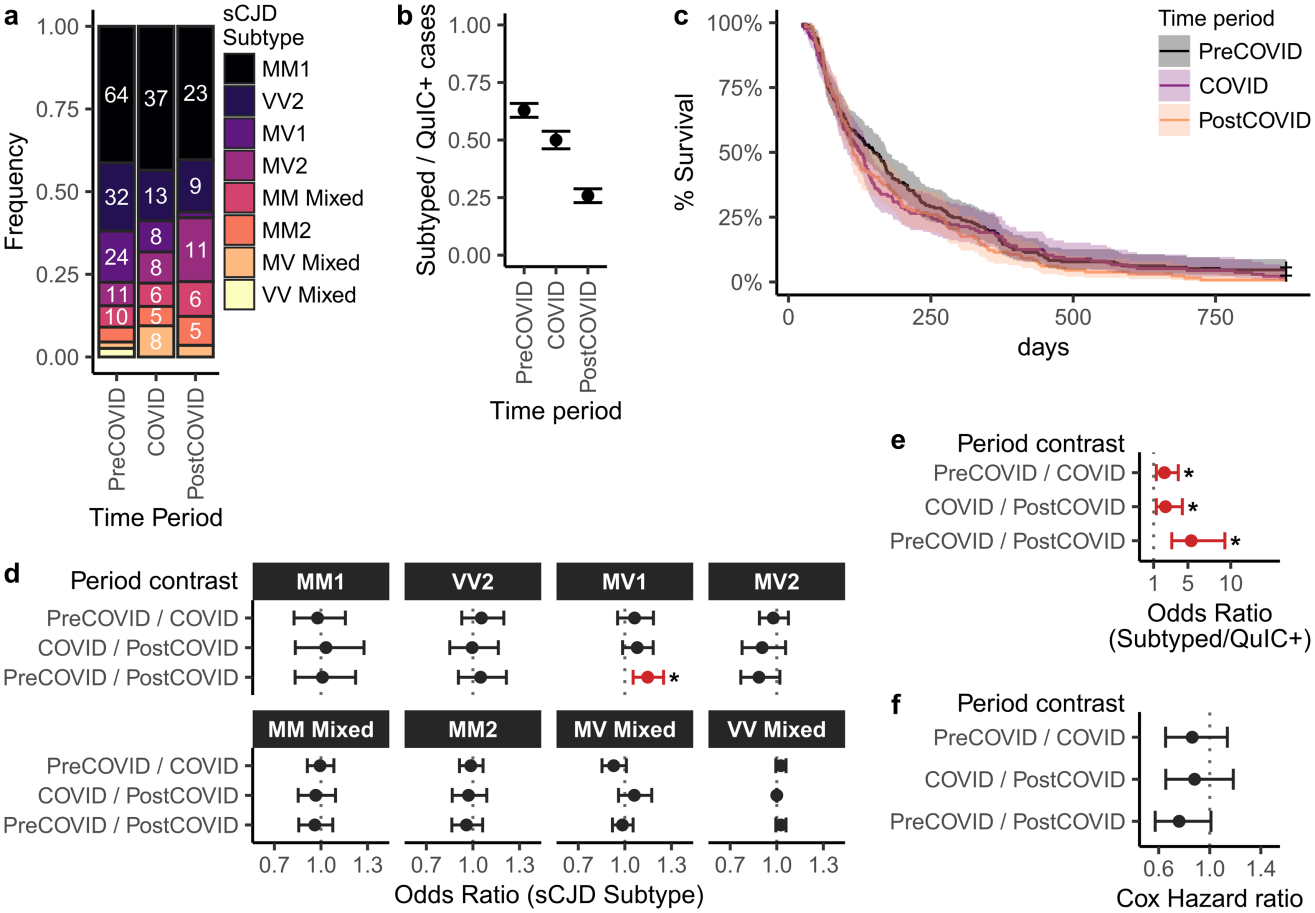
Comparison of sCJD subtype distributions and disease durations in Canada across pre-COVID, COVID, and post-COVID periods. Shown are **(a)** distribution of sCJD subtypes, **(b)** subtyping rates among EP-QuIC positive cases, and **(c)** Kaplan–Meier survival curves of disease durations. Panels **(d–f)** show pairwise comparisons between study periods for **(d)** subtype distributions (multivariate logistic regression), **(e)** subtyping rate (binomial logistic regression), and **(f)** disease duration (Cox proportional hazards ratios). Estimates are shown with 95% confidence intervals. * *p* < 0.05. Pre-COVID = 2016-01-29 – 2020-02-28, COVID = 2020-03-01 – 2022-09-30, post-COVID = 2022-10-01 – 2025-09-29.

sCJD can be classified into molecular subtypes based on the combination of *PRNP* codon 129 genotype (MM, MV, or VV) and electrophoretic profile of proteinase K-resistant PrP (type 1 or type 2), yielding six subtypes: MM1, MV1, VV1, MM2, MV2, and VV2 (22, 23). To assess whether CJD characteristics changed during the pandemic, we compared molecular subtype distributions across the pre-COVID, COVID, and post-COVID intervals in a subset of EP-QuIC-positive cases that underwent post-mortem examination (Figures 3a,d). Across all periods, the most prevalent subtypes were sCJD MM1 and VV2, which is commonly seen.

Multinomial logistic regression revealed no significant differences in the subtype prevalence between the pre-COVID and COVID eras. However, sCJD MV1 prevalence was significantly lower in the post-COVID period (1/57) compared to pre-COVID (24/155; *p* = 0.0016), but not COVID (8/85; *p* = 0.11) intervals. The cause of this decline is unclear, although it may reflect SARS-CoV-2 exposure, methodological changes in subtyping protocols, or simply the sporadic nature of CJD.

Differences in subtyping coverage across study periods could confound our analysis of subtype distributions. To assess this, we examined the proportion of EP-QuIC positive cases in each period that subsequently were subtyped (Figures 3b,e). Subtyping frequency declined during COVID and further in the post-COVID period. The cause of this decline is unclear, although potential reasons include the delay between QuIC-detection and subtyping (mean = 321 days), reduced enrollment in the national autopsy program, and pandemic related restrictions. Nevertheless, underrepresentation of subtyped post-COVID cases may have influenced the observed MV1 prevalence trends.

We also investigated whether CJD disease durations changed during the pandemic. Dates of symptom onset and death were available for 193 pre-COVID, 136 COVID, and 131 post-COVID sCJD cases. Disease durations, defined as the time difference between symptom onset and death, were compared using Kaplan–Meier survival curves and Cox proportional hazards ratios (Figures 3e,f). Disease durations were right-censored at 874 days to match the post-COVID maximum, excluding 11 cases with unusually long durations. Median disease durations were 150 (pre-COVID), 126 (COVID), and 113 days (post-COVID), with no significant differences (Figures 3c,f). However, this analysis is limited by the relatively small number of cases and the potential for longer-duration subtypes (e.g., MM2) to be underrepresented in the post-COVID interval.

While a decline in MV1 subtype prevalence was observed post-COVID, we concluded that overall sCJD subtype distributions and disease durations were largely comparable across the study periods.

## Discussion

By surveying EP-QuIC positive CJD cases, we found that Canadian CJD incidence remained stable throughout the COVID-19 pandemic, despite a 12% increase in test submissions. Although defining the COVID period by the removal of restrictions might oversimplify the gradual return of healthcare services, the lack of association between EP-QuIC positive tests and SARS-CoV-2 cases suggests that CJD incidence was stable independently of the chosen dates. Thus, the consistency of CJD prevalence and the temporary testing surge suggests that Canadian CJD diagnostics and surveillance was unimpeded during a period of healthcare disruptions.

Mechanistically, COVID-19-associated neuroinflammation (13) and the amyloidogenic properties of SARS-CoV-2 proteins (14–17) are plausible links to prion misfolding. However, CJD’s rarity and long pre-clinical incubation periods (10 to 40 years) (24) makes it very challenging to assess epidemiologically whether SARS-CoV-2 is a prion disease risk factor. Consistent with findings from the United Kingdom (18), Germany (19, 20) and Australia (21), here we did not observe an increase in CJD incidence during the pandemic. Nevertheless, long-term monitoring may identify any effects of SARS-CoV-2 exposure on CJD prevalence in the years to come.

It has also been theorized that COVID-19’s neuroinflammatory effects (13) could accelerate CJD progression. While some studies argued in favor of this hypothesis (25, 26), surveillance data from the United Kingdom (18) found no association between COVID-19 status and CJD duration. Similarly, our analysis did not reveal significant differences in sCJD duration before, during, or after COVID-19. However, this conclusion was limited by the relatively small number of cases that could be linked with dates of death and symptom onset. The effect of COVID-19 on CJD disease duration may be more definitively assessed by comparing larger cohorts of SARS-CoV-2 exposed and non-SARS-CoV-2 exposed CJD patients.

The observed post-pandemic decline in sCJD MV1 prevalence is interesting but difficult to interpret. It may reflect the continual decrease in autopsy rates, changes in subtyping protocols, or natural variation in sCJD subtype distribution. Given that MV1 and MM1 are often clinically indistinguishable and grouped under “classical CJD” (27), missed diagnoses due to atypical presentations are unlikely. Moreover, *PRNP* codon 129 genotype distributions remained consistent across all periods, ruling out population-level genetic shifts as a contributing factor. Continued monitoring of sCJD subtype distributions may determine whether the decline in MV1 prevalence is sustained.

Collectively, our findings suggest that COVID-19 did not influence the incidence or characteristics of CJD in Canada. Our study also highlights the value of ante-mortem EP-QuIC CSF testing in monitoring CJD prevalence, which may evolve in response to shifting population demographics or environmental exposures.

## Data Availability

The raw data supporting the conclusions of this article will be made available by the authors, without undue reservation.

## Footnotes

1
https://doi.org/10.25318/1710000501-eng
